# What Does a Hand-Over Tell?—Individuality of Short Motion Sequences

**DOI:** 10.3390/biomimetics4030055

**Published:** 2019-08-07

**Authors:** Holger H. Bekemeier, Jonathan W. Maycock, Helge J. Ritter

**Affiliations:** 1 Neuroinformatics Group, Bielefeld University, 33615 Bielefeld, Germany; 2 Margin UG, Goldstraße 9, 33602 Bielefeld, Germany

**Keywords:** hand-over, individuality, motion capture, neural network

## Abstract

How much information with regard to identity and further individual participant characteristics are revealed by relatively short spatio-temporal motion trajectories of a person? We study this question by selecting a set of individual participant characteristics and analysing motion captured trajectories of an exemplary class of familiar movements, namely handover of an object to another person. The experiment is performed with different participants under different, predefined conditions. A selection of participant characteristics, such as the Big Five personality traits, gender, weight, or sportiness, are assessed and we analyse the impact of the three factor groups “participant identity”, “participant characteristics”, and “experimental conditions” on the observed hand trajectories. The participants’ movements are recorded via optical marker-based hand motion capture. One participant, the giver, hands over an object to the receiver. The resulting time courses of three-dimensional positions of markers are analysed. Multidimensional scaling is used to project trajectories to points in a dimension-reduced feature space. Supervised learning is also applied. We find that “participant identity” seems to have the highest correlation with the trajectories, with factor group “experimental conditions” ranking second. On the other hand, it is not possible to find a correlation between the “participant characteristics” and the hand trajectory features.

## 1. Introduction

### 1.1. Information Content of Short Motion Sequences

In this study, we are interested in the information content of short motion sequences, especially on individuality. There are many examples of willfull transport of information by motions, like finger spelling. An interesting technical approach in this field was presented by N. J. Kuch in 1989 [1].

However, there is also a vast amount of information about identity and personal properties, which is transported via motion unknowingly by the performer.

There are several studies dealing with this type of information (for comparison, see the subsection “Related Work”). Our approach is the use of hand-over motions to derive information about the individuals performing the motions or the experimental conditions. The benefit of hand-over motions is the possibility of an exact definition of the conditions and the task. In addition, it is a kind of motion, which is not evident as a method of communication as it is primarily used for another, practical use, namely the handling of objects. This means that our study has a strong focus on the question: how much information about the performing person and the experimental condition is transported unwillingly.

Hand-over motions are a class of movements, which can be subdivided in different types of motion, which occur in everyday situations, and are well-practiced. This suggests using them in a study which focusses on unwilling information transport. As a side-effect, a study like this could also help to enhance the performance of robots to assess intended motions of human partners, as the information content of human movements has to be known for this.

Short motion sequences are potentially rich sources of information, for example about the experimental conditions, under which they were performed, or the individuals, who performed them. Do similarities between motion sequences allow conclusions, what commonalities exist between all trajectories, which were performed under the same conditions by the same individual or by an individual with similar properties? In the case of formerly unknown trajectories, is it possible to identify the conditions, the individual or the properties of the performing participant just by an automatic classification, after training a suitable neural network? We studied these questions for the example of the human hand-movement, to be more specific, for the hand-over of objects between two participants.

### 1.2. Potential Factors Identifiable by Trajectories

One possibility is that differences and similarities between the motion sequences are related to differences and similarities between the individuals who performed the motions, to their identity, or to the experimental conditions, under which the motions were performed, so that the observed trajectories allow conclusions about the properties of the individuals, their identity, or properties of the performed trial. The participants differ in physical, cultural, interpersonal and psychological factors. In detail, we are interested in identity, gender, age, body size, weight, profession, sportiness and citizenship of the persons. Further factors, such as the time since the last meal, the familiarity of each subject with his or her experimental partner and the medical history of the participants could have influence on the hand-over movements.

The given task [2], the identity of the participant [3,4,5,6,7,8,9,10,11], his or her properties like gender [12,13,14,15], body height and weight [16], or movement disorders [17] were already found in several studies to be factors, which can be identified by evaluating motion trajectories. In addition, the influence of age, psychological factors, occupation, ethnicity or the nutritional status on motoric observables like grip strength is already identified. [18] The influence of practice on motorical skills is also well-known [19]. More details on other studies are given in the subsection “Related Work”.

**Figure 1:**
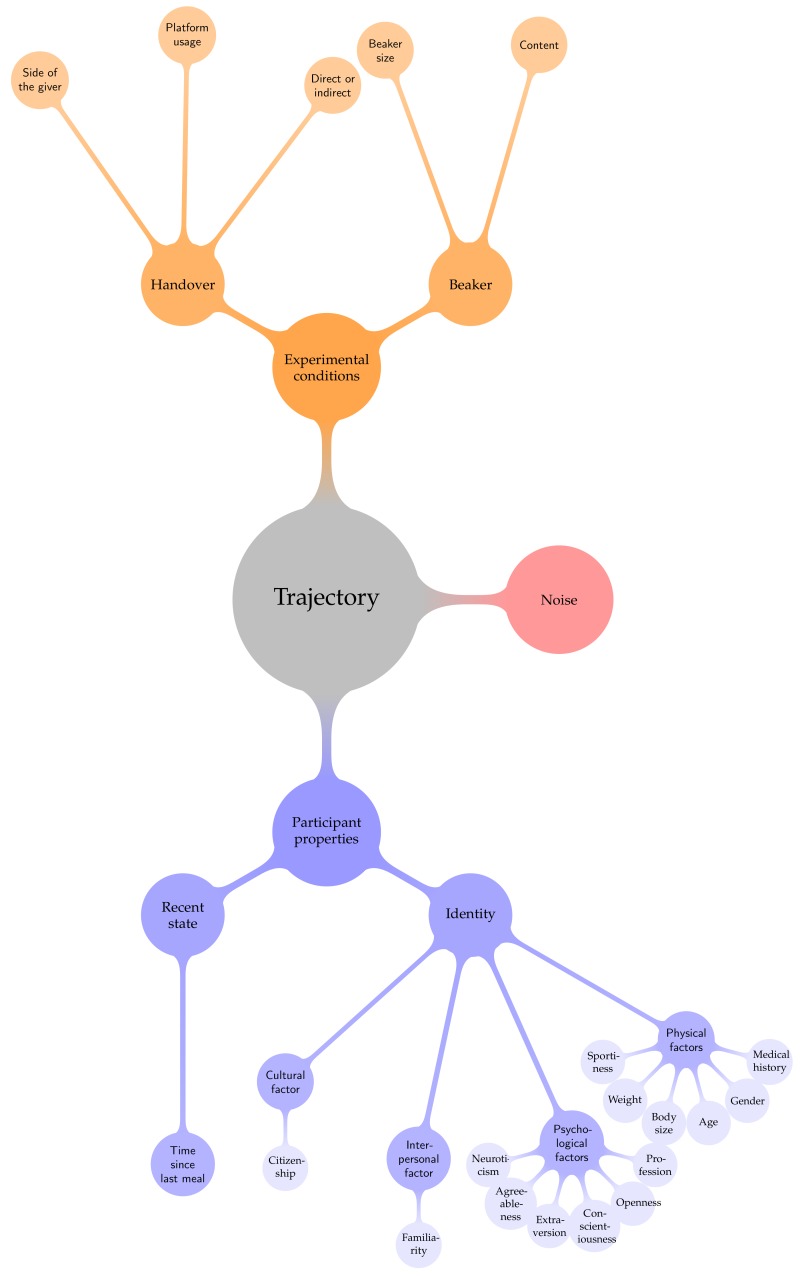
Tentative hierarchy of the factors, which could have an influence on the trajectories.

All of the mentioned factors are different properties of the participants. Under given experimental conditions, is it possible to determine the participant, who performed the motion or some of his or her properties by an automatic classification of the trajectory? As mentioned in the preceding subsection, the experimental conditions are further factors, which could be determined in the same way. Before the experiments, the properties of the participants were assessed. Then, we studied if there is a statistically significant correlation of factors like the experimental conditions, the participant identity or the participant properties and the hand trajectories, or if the correlation between these factors and the trajectories is overshadowed by random fluctuations. The studied factors and the possible connections between them are sketched in Figure 1.

The asked question can be reformulated in more detail as follows:

1. When the participants or the experimental conditions are similar or the same, do the trajectories also show similarities, so that a matching between the trajectories and factors like the participant identity or the experimental conditions is possible?
2. Do the trajectories have different properties, indicating distinguishable behaviours, if the experimental conditions or the properties of the participants are different? Is there a statistically significant relation between a classification of the trajectories on one side and differences between the persons and the experimental conditions on the other side?
3. Can we find a hierarchy of similarity in factors like the experimental conditions, the participant identity or the participant properties, which is mirrored by a hierarchy of similarity of the trajectories, so that the trajectories are more similar, if the experimental conditions or the properties of the participants are more similar? What hierarchy level do the factors or factor groups “individuality”, “participant properties” and “experimental conditions” have, when we ask for their correlation with the hand trajectories? Which factor has a higher or lower correlation? What shapes a hand-over motion most, what shapes it least?

An additional interesting question, which could be answered in the described way, is how stereotypically hand-over motions are performed, by the same participant under constant external context conditions. Are the differences between the trajectories significantly smaller, if these factors are kept constant, in comparison to the differences between trials with different conditions or different participants?

### 1.3. Experimental Design

To answer these questions, it was essential to use a set of well-defined, repeatable experimental conditions, which was the same for all participants. The following considerations lead to a suitable set.

To get comparable conditions for the chosen hand-over scenario, at first, we needed standardised test objects which are similar to objects used in everyday life. We used plastic cans with a constant diameter. To generate differences in the free space, which is available for the participants to apply a grip to the beaker, two different beaker heights were used. To induce further differences in the grasp-behaviours, we used both filled and empty cans. When filled, the beakers have a much higher weight (790 g in the case of the high, and 535 g in the case of the low beakers) than the empty ones (40 g in the case of the high and 35 g in the case of the shorter beakers). It could be that different persons behave differently in the same one of both roles (giver or receiver) because of individual differences and differences in the position. Because of this, in one experiment, both participants sometimes served as the giver and sometimes as the receiver. The degree of necessary information transport between the participants depends on the task. If an object is handed over directly from the hand of the giver to the hand of the receiver, the latter needs more information about the performed, ongoing and intended motions of the giver than in the case when the object is put down and released by the giver, before the receiver grasps for it; in this case, the receiver only needs information about the position of the object itself, not about the motions of the giver, who placed it there. This is one reason why we expect completely different grasp-behaviours, when we test both direct and indirect hand-over. We also expect that two people could try to avoid direct contact to the other participant, when they both have to touch the object at the same time, depending on their psychological and interpersonal properties. Other differences, which can be tested, are those between hand-over motions in different heights or those between hand-over motions with more or less restricted space. For this purpose, we introduced a platform that can be placed between the participants in a part of the trials or left out in other trials. A small wooden rack can be used for this.

### 1.4. Feasible Motion Primitives for Artificial Systems

As we have a number of factors, in which both the experimental conditions and the participants differ, we expect to get motion trajectories, which also differ in many aspects. In addition, the mechanically possible solutions for the human hand to solve a single one of all given tasks can be seen as nearly infinite. On the other hand, the resources of the nervous system are limited. Evolution tends to reduce nervous tissue to the necessary minimum, as the energy consumption of neurons is relatively high. This allows the question of if the high number of all possible combinations of muscle activations to solve a given task is reduced in reality to a much smaller set of co-activations with a much lower redundancy. This would mean that the same participant in the same experimental situation could react with similar motions, based on a similar sequence of co-activations which could be addressed as motion primitives, as the activation of the nervous and muscular system could also be similar. We try to find a stereotypy like this in the reactions of our participants on a given situation. If this stereotypy is distinct enough, it could be possible to determine the underlying motion primitives. These could be transferred to an artificial system like the shadow hand (see, for example, Röthling et al. [20]) in further studies. The experiment described here is restricted to the biological side of the question.

### 1.5. Related Work

Other studies have already shown that it is possible both for humans and for artificial systems to derive information about the identity or properties like the gender of people or the task they had to perform, from their movements, e.g., from their head and facial motions (human observers: Hill and Johnston [12], Girges et al. [10]), from their eye motions (artificial identification: Bednarik et al. [6]), from their gait (human observers: Cutting and Kozlowski [3], Troje et al. [8], artificial identification: Little and Boyd [5], Lu et al. [15], comparison of human and artificial identification: Troje [13]), from traditional Japanese dance sequences (artificial identification: Perera et al. [2]), or from a database with different types of motions (human observers: Loula et al. [7], database for artificial identification: Ma et al. [14]).

Human movements can be used to identify the person, who performed them, and can even be used as part of an authentication system (Cunado et al. [4], BenAbdelkader et al. [16], Green and Guan [17], Bednarik et al. [6], Lin et al. [9], Neverova et al. [11]). A high correlation between individuality and the characteristics of hand-over motions in our study could show that also hand-over can be used as a kind of motion finger print.

Some of the methods we use are also applied in some of the mentioned studies, like the data acquisition via motion capturing (e.g., Hill and Johnston [12], Girges et al. [10], Perera et al. [2], Ma et al. [14], Lin et al. [9]) or the classification by a Self-Organizing Map (Lin et al. [9]).

For example, how knowledge about non-verbal communication can be used in robotics was presented by Hameeteman [21]. He tried to enhance the acceptance of robots by equipping them with more human-like movements. The motion sequences collected in our study could also serve as examples of natural human motions for the same purpose as a side effect. We are focussing on interactive motions, and, in this case, it can be very helpful to equip an artificial system with human-like motions, as this enhances the predictability of the movements and therefore the efficiency of the interaction.

## 2. Materials and Methods

### 2.1. Participants

Our study is performed with human participants. For one experiment, two persons are needed, as hand-over motions between both are analysed.

The participants differ in their properties, namely physical, cultural, interpersonal and psychological aspects. In more detail, we are interested in identity, gender, age, body size, weight, profession, sportiness and citizenship of the subjects. Concluding the identity of a participant from the trajectories means uniquely identifying individual persons from their motions. In addition, the time since the last meal, the familiarity of each participant with his or her experimental partner and the health state of the participants could have an influence on the hand-over movements. To get information about the mentioned properties, the subjects had to fill out a query. The results are listed in Table 1. “Sportiness” is measured as the time for doing sports in one week. “Satiety” is measured as the time since the last meal of the participant. “Familiarity” means the familiarity of the subject with the other one, assessed on a scale from 1 (don’t know) to 5. For the factor “health”, the answer to the question, if there are any problems with arms, shoulders or hands, is given. As all analysed participants have the same citizenship, this factor is dropped in the analysis.

For the example of grip strength, Lee et al. [18] have discussed an influence of psychological factors on motoric phenomena. We are also interesed in finding an influence of psychological factors on the motoric behaviour of our participants. Therefore, we are assessing the so-called “Big Five personality traits”. This method advanced to a standard approach in many psychological studies, as John et al. discussed in 2008 [22]. Correspondingly, our participants had to perform a Big Five Test. We chose the test designed by Jeff Potter available online [23]. He used data from that project for a high number of psychological research publications (e.g., as co-author of Srivastava et al., Robins et al., Carney et al., and Soto et al. [24,25,26,27]). The results of the test are five factors with values between 0 and 100, namely “openness” (to experience), “conscientiousness”, “extraversion”, “agreeableness” and “neuroticism”. The factors for the different participants (P) of our study are given in Table 2.

### 2.2. Setting

**Figure 2:**
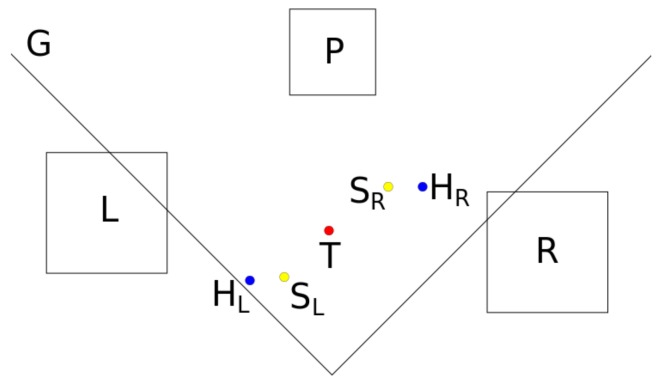
Schematic diagram of the experimental setup, seen from above. *G* is the glass plate, *L* the stool of the left participant, *R* the stool of the right participant, *P* the wooden rack, which can be used as platform, $H_{L}$ and $H_{R}$ the resting places for the right hand of the left or right participant, respectively, $S_{L}$ and $S_{R}$ the start and destination positions of the plastic can, which can change their roles together with the role of the participants, and *T* the place of the hand-over.

**Figure 3:**
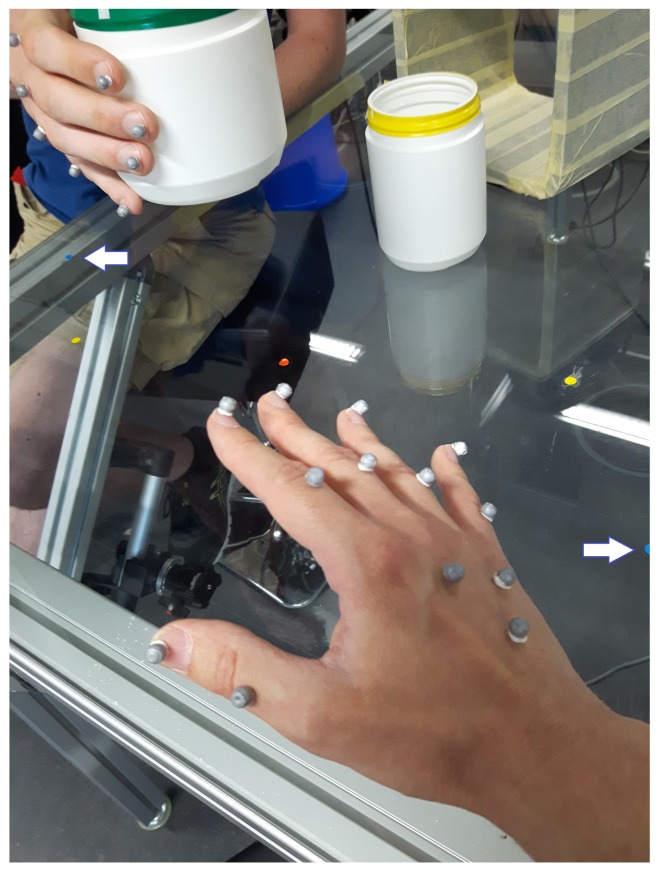
Photo of the experimental situation, seen from the position of the participant sitting on stool *R*. (Denotations as in Figure 2.) His hand with 13 reflective markers is visible in the lower center. The low number of markers is possible because of the application of inverse kinematics when determining the joint angles—see Maycock et al. [28]. The participant on stool *L* is visible in the upper left, the rack *P* in the upper right, one of the plastic cans standing in front of it. (Just for visualisation, normally, the cans are stored on an extra table behind the experimenter.) The points $H_{L}$ and $H_{R}$ are visible as small blue disks close to the left or right image border, respectively. (For better visualisation, white arrows pointing to $H_{L}$ and $H_{R}$ were added.) The points $S_{L}$ and $S_{R}$ are the small yellow disks next to $H_{L}$ and $H_{R}$, respectively. Point *T* is the small red disk between them, close to the center of the picture. The situation of a direct hand-over of a small, empty can in the moment is shown, when the giver on stool *L* is holding the can over point *T*, and the receiver on stool *R* reaches for the can. (This would be an example of an incorrect behaviour of the participants, as a green border of the can signalises an indirect hand-over. Trials like this were sorted out. In the experiment, markers were also put at the upper border of the cans.)

The participants sit on stools (*L* and *R* in Figure 2) on two sides of a glass-plate (*G*) serving as a rectangular table, the sides with the persons sharing a corner. (The setting is also shown in Figure 3). A big cuboid metal rack serves as an attachment for the glass plate and the recording cameras.

**Figure 4:**
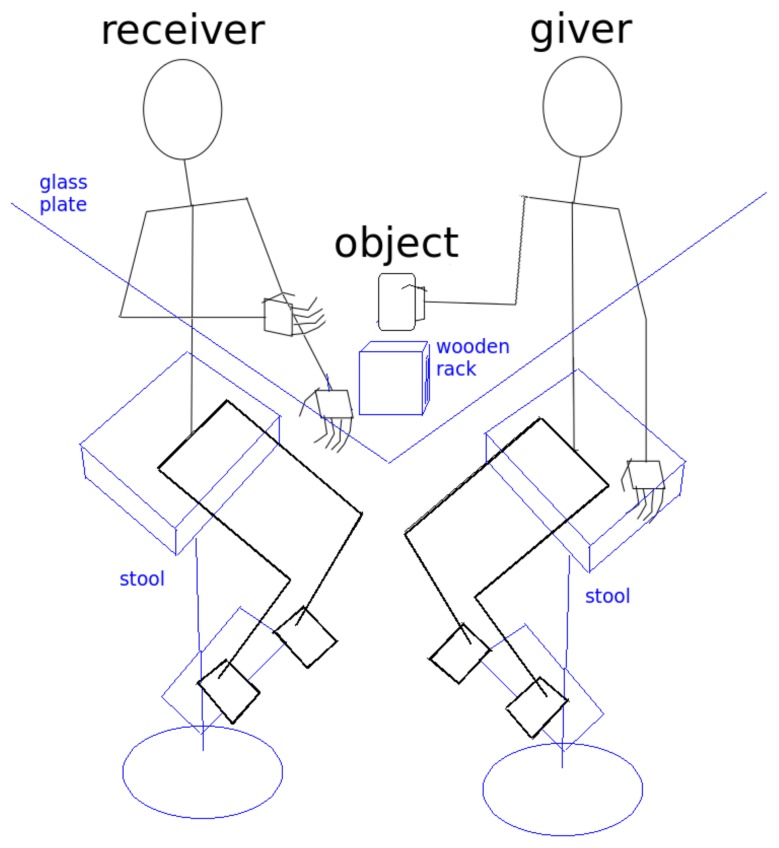
Sketch of the experimental setup, seen from the position of the experimenter.

A small wooden rack (*P*) can be placed at the point *T* between the participants, as it is shown also in Figure 4, its two closed sides pointing to the persons. The rack can be removed by shifting backwards. (This situation is shown in Figure 2 and Figure 3.) If the platform is used in a trial or not is one factor defining the experimental condition.

The participants have to hand-over a plastic can at or over the point *T* between them, which is marked visibly for them. We used cans of two different heights (141 mm and 103 mm) with equal diameter (100 mm). The beaker size is another factor defining the experimental condition.

The beaker is either empty or filled with stained water. This difference defines a third factor of the experimental condition. When they are empty, the higher cans have a weight of 40 g and the shorter cans have a weight of 35 g. When filled, the higher cans have a weight of about 790 g and the shorter cans have a weight of about 535 g, as mentioned above.

The colour of the border of the can gives the information to the participants, if they have to perform a direct (yellow border) or indirect (green border) hand-over. In the case of a direct hand-over, the can is handed over from the giver to the receiver over point *T* without touching the glass plate or the wooden rack, after it was taken from the starting point ($S_{L}$ or $S_{R}$) and before it was placed at the destination location ($S_{R}$ or $S_{L}$). ($S_{L}$ and $S_{R}$ change their roles as starting point or destination location depending on the role of the participants. If the left person is the giver, $S_{L}$ is the starting point, and $S_{R}$ is the destination location; if the right person is the giver, $S_{R}$ is the starting point, and $S_{L}$ is the destination location.) In the case of an indirect hand-over, the giver puts the object down at the hand-over point *T*. When the platform stands at point *T*, the object is put onto it, otherwise the can is put directly onto the glass plate. The difference between a direct and an indirect hand-over symbolised by a yellow or green border, respectively, is a fourth factor of the experimental condition.

Both participants stay at their places *L* and *R* in the entire experiment, but they change their roles as giver or receiver depending on the trial, leading to different geometrical situations, as both persons are asked to use only their right hand for handing over the can. The two possible choices of the subjects as giver or receiver are a fifth factor defining the experimental condition.

We are testing all combinations of the five binary valued factors platform usage (with or without rack), beaker size (big or small), border colour of the can (green or yellow), content of the beaker (full or empty) and side of the giver (right or left participant). As there are $2^{5}$ = 32 combinations of the five factors with two possible values for each, we get 32 different experimental conditions. We decided to allocate three trials to each of these 32 conditions, leading to a total number of 96 trials. The sequence of the trials was randomized, so that the 32 conditions appeared in an arbitrary order.

### 2.3. Experimental Procedure

Always one participant with an odd number and the following one are experimental partners in the same experiment.

Before the experiment, the right hand of the left person rests at point $H_{L}$ in Figure 2 and the right hand of the right subject at point $H_{R}$. Both points are marked visibly for the participants. The experiment starts with placing one of the plastic cans at a fixed starting place, also marked visibly, close to the hand of the person chosen to be the giver. If the left participant is the giver, $S_{L}$ is the starting place; if the right subject is the giver, $S_{R}$ is the starting place. The giver takes up the object and moves it to the hand-over point *T*. (This moment is shown in Figure 3). The receiver takes the object and puts it at a pre-defined destination location. If the left participant is the giver, $S_{R}$ is the destination location; if the right subject is the giver, $S_{L}$ is the destination location. In other words, $S_{L}$ and $S_{R}$ change their roles as starting point and destination location depending on the roles of the participants.

The right hands of the participants, which are used for the hand-over, are recorded using motion-capture to get time-courses of their finger limbs in the hand-over process.

### 2.4. Measured Values

The kinematic data are acquired as time courses of three-dimensional positions of markers attached to the different finger limbs of both participants.

For recording the right hand of both participants, we placed one marker at the tip of each finger nail, one marker slightly distal of each knuckle (metacarpo-phalangeal joint) and three markers near the center of the back of the hand (metacarpus), one of these proximal of the center, two distal of the center, as it is shown in Figure 3. This leads to a total of 13 markers at the right hand of each participant.

We did not place markers proximal to the distal interphalangeal joints of the fingers or proximal to the matacarpo-phalangeal joint of the thumb. In a classical approach, this would be necessary to calculate all relevant joint angles. However, our model-based approach to estimate the joint angles uses inverse kinematics. This means, a realistic hand posture is calculated from the marker data by using a hand model which is fit to the marker positions. With our reduced number of markers, several joint angle combinations could lead to the same marker positions, but our approach solves this redundancy problem by inverse kinematics. Classical approaches with more markers cause problems with marker occlusions and ghost markers, leading to less accurate results. Because of this, we used the approach, which was developed by Maycock et al. [28].

At each of the beakers, three markers were attached in a plane at the circular upper border in an angular distance of approximately 90${}^{\circ }$. When the plane is seen from above and the beaker turned in an appropriate way, one marker is visible on top, one on bottom and one on the left side. In this perspective, a fourth marker is placed close to the upper one, but below the plane of the other three markers, so that the markers can be used to form a three-dimensional coordinate system. To sum it up, a total of 30 markers has to be tracked in each trial.

To record the positions of the markers attached to both participants and the object over time in three dimensions, we use a VICON system from Prophysics AG (Kloten, Switzerland), which observes them with a multitude of 17 cameras out of different angles. The exactness of the recording is in the range of 0.5 mm. The system is calibrated at the beginning of each experimental day. For the calibration, the sensitivity of the single cameras is optimized, disturbations from the infrared lights of other cameras and reflections are masked, and a so-called “wand” with five markers in a rectangular arrangement is moved in the relevant area to train the three-dimensional detection by the system.

### 2.5. Data Analysis

VICON was used to track the markers and, to ensure a robust and efficient labelling was performed, the Auto Tracker method developed by Maycock et al. [28] was employed. This fits models into the scene— in our case, models for the two right hands and the beaker, and uses an inverse kinematics approach to minimise the distance error to each marker. This allows for hand joint angle values to be computed and extracted. To ensure accuracy and in order to take account of hand size differences, a normalisation step was performed.

We assume each hand as containing joints with a total of 20 rotational degrees of freedom, which are relevant for our study. Namely, in the view of our slightly simplified model, each finger, including the thumb, has one basal joint with two degrees of freedom and two more distal joints each with one degree of freedom, leading to a number of four degrees of freedom for each finger and a total of 20 rotational degrees of freedom per hand.

The values of these 20 angles were calculated by the Auto Tracker for each time step. The degrees of freedom for the translation and rotation of the entire hand are not respected in this study, as it can not be expected that they are similar in different trials, even not for the same participant under the same conditions, as the absolute position of the person is not fixed, so that the translatory movement of his or her hand is influenced strongly by random fluctuations from trial to trial.

We used Dynamic Time Warping for measuring the distances between all time courses of the joint angles. Next, the distances contained in the resulting similarity matrix were evaluated via multidimensional scaling. The multidimensional scaling applied on the similarity matrix was performed as nonmetric multidimensional scaling with Kruskal’s normalized stress1 criterion. This criterion is the stress normalized by the sum of squares of the inter-point distances.

We used the function “mdscale” of MATLAB’s Statistics and Machine Learning Toolbox (MATLAB version R2018b, The MathWorks Inc., Natick, Massachusetts, USA) to perform the scaling. Our choice of the parameters is given in Table 3; the names of the parameters are explained in the online help [29].

The further analysis of the data was performed by use of neural networks in two different ways. We applied both supervised learning, as it is described in the next subsection, and unsupervised learning, as it is described in the next but one subsection, to train the network.

### 2.6. Supervised Learning

A Learning Vector Quantization Network [30] was fed with the positions of the projections of the trajectories and trained to recognize the value of one of the experimental factors by the trajectory.

In other words, after the training, the network should be able to classify the trajectories into groups. To test this, if the network is able to classify also formerly unknown trajectories correctly after the training in the described way, a Cross Validation was performed. The division of the dataset for the Cross Validation was performed in two different ways.

“Trialwise”: Trials from all participants were assigned arbitrarily. “Participantwise”: In this case, each partition contains all trials of only one participant. With this method, it can be tested if the network is able to recognise the value of a participant property for a trial of one participant, when it was only trained with trials of other participants.

For all factors, the mutual information was calculated by use of the function “information” presented in [31]. We left the descriptor unspecified in a first step. Then, the determination of the mutual information was repeated in the sense of an N-unbiased estimate with the descriptor found in the first step and the base two for the logarithm. When calculating the mutual information of the trajectory and other factors, the Multidimensional Scaling was performed in the sense of a projection into spaces with different dimensionality, so that the trajectory is represented by a number of factors corresponding to the dimensionality.

### 2.7. Unsupervised Learning

If we want to find a connection between the experimental conditions, the participant identity, and his or her properties on one side and the position of the projections of the trajectories in the projection space on the other side by analysing the clustering in this space, we can also use unsupervised learning. One possibility is the use of a Self-Organizing Map [32]. This is the option we chose. The map was trained to recognise the positions of the projections of the trajectories in the projection space. The training process was performed in that way that the weight positions of the map corresponded as good as possible to the clustering of the positions of the projections of the trajectories in the projection space afterwards. After the training, only one neuroid of the map gave a non-zero output, when the positional data of the projection of a new hand trajectory was fed into the map.

In addition, in the case of unsupervised learning, Cross-Validation was applied, so that the trajectories, which were used for testing the map, were not contained in the training subset. When testing the map, the position of the neuroid, which gave a non-zero output, was determined. We used an eight-dimensional map with a hexagonal architecture. Along each of the eight dimensions, the map contained two neuroids, which leads to a total of $2^{8}$ = 256 neuroids. In an eight-dimensional map, the neuroid position is defined by row, column, plane, cube, tesseract, penteract, hexeract and hepteract. For each trajectory, these eight numbers were determined for the respective neuroid giving a non-zero output. A statistically significant relation between experimental factors and the neuroid position would indicate a correlation between these factors and the hand trajectory. The experimental conditions, the participant identity and his or her properties form a set of independent variables, whereas the eight numbers, which define the neuroid position, form a set of dependent variables.

A suitable statistical method to test the relation between a set of independent variables on a set of dependent variables is a MANOVA. The set of independent variables can be reduced to two, namely the number of the experimental condition and the number of the participant. This was done in a first statistical test.

## 3. Results

### 3.1. Multidimensional Scaling

**Figure 5:**
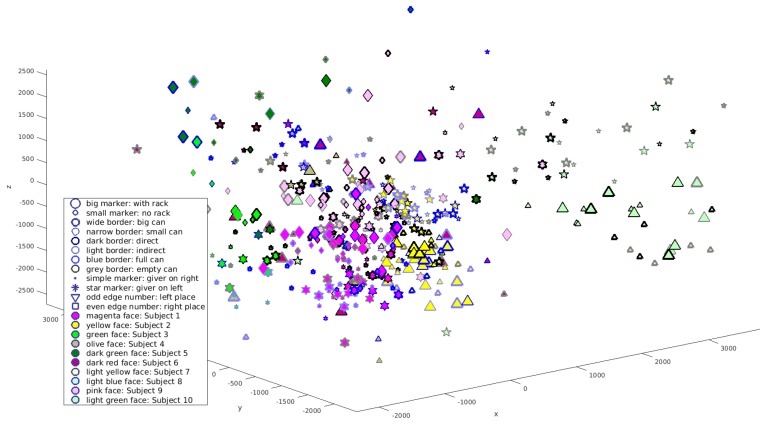
Results of the multidimensional scaling into three dimensions for all participants except “Subject 7” and “Subject 8”. The axes of the three-dimensional projection space are labelled with *x*, *y* and *z*. Each trajectory is represented by one marker in this space. Different experimental conditions are symbolized by different marker limits, and different participants by different colours of the markers, as described in detail in the legend.

The result of the multidimensional scaling for three dimensions is given in Figure 5. When the Auto Tracker fits the hand models to the data, it can happen that it confuses the right hands of the two participants with each other. It failed in this way in many trials of subjects 7 and 8. Because of this, the data of these persons are not shown in Figure 5 and not used for the further analysis. The problem did not occur or occurred very seldomly for the other participants.

In Figure 5, markers with the same colour represent the same participant and star-shaped and convex markers represent the roles of each person as giver or receiver, respectively. As can be seen from the distribution of markers with different colours in the tigure, hand trajectories of different participants are concentrated in different regions of the space, separated from the trajectories of other people. In addition, for each participant, markers belonging to the role as giver or receiver, respectively, are concentrated in different regions and separated from each other.

**Figure 6:**
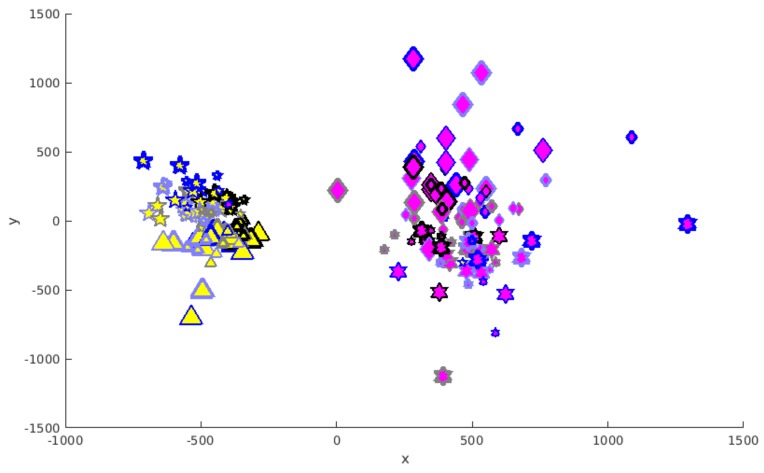
Results of the multidimensional scaling into three dimensions for the participants “Subject 1” and “Subject 2”. The axes of the three-dimensional projection space are labelled with *x* and *y*. The *z*-axis is pointing to the viewer. Use of markers equivalent to Figure 5, legend see there.

The distances between the trajectories were additionally calculated for the two interacting partners in a single experiment without respecting the trajectories of other experiments and then projected via multidimensional scaling into the three-dimensional space. As an example for this, the results for the experiment with participants 1 and 2 are shown in Figure 6. The *z*-axis of the projection space is not visible here, as it is pointing to the viewer.

As can be seen from the distribution of markers with different colours, the markers for the two participants form two distinct clusters, both consisting of two non-intersecting subclusters for the roles as giver or receiver (star-shaped and convex markers, respectively). Equivalent observations can be made also in the case of the other single experiments (not shown here). Markers for trials with empty cans are concentrated in the central area—those for a full container can also be found at positions farther away.

**Figure 7:**
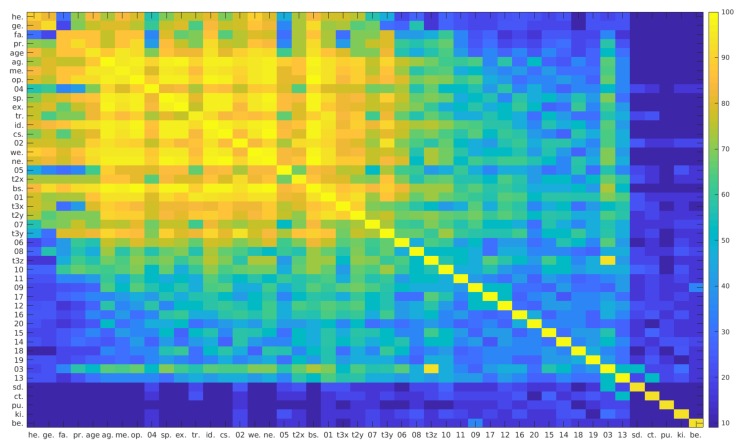
Mutual information between the experimental factors and the trajectory. The different factors including the trajectory are given on both axes, the mutual information of two factors is represented by the colour at the intersection of the row and column belonging to the two factors. The colour coding is given on the bar on the right side. The numbers on the bar represent percentiles of all mutual information values. The different factors are abbreviated in the following way: he.: problems with the right arm or shoulder, ge.: gender, fa.: familiarity with the other participant, pr.: profession, age: age of the participant, ag.: agreeableness, me.: time since last meal, op.: openness, sp.: hours for sports per week, ex.: extraversion, tr.: one-dimensional projection of the trajectory, id.: identity of the participant, cs.: conscientiousness, we.: weight, ne.: neuroticism, t2x: *x*-component of the two-dimensional projection of the trajectory, bs.: body size, t3x: *x*-component of the three-dimensional projection of the trajectory, t2y: *y*-component of the two-dimensional projection of the trajectory, t3y: *y*-component of the three-dimensional projection of the trajectory, t3z: *z*-component of the two-dimensional projection of the trajectory, sd.: side of the giver, ct.: content of the beaker (full or empty), pu.: platform usage, ki.: kind of handover (direct or indirect), be.: beaker size. The numbers at the abscissa and the ordinate are referring to the components of the 20-dimensional projection of the trajectory.

The results for the analysis of the mutual information are shown in Figure 7.

The mutual information between the experimental conditions and the other factors except the trajectory (one-, two-, three-, and 20-dimensional) is always close to zero; a statistical correlation between experimental conditions and hand trajectory can be shown in this way, but not between experimental conditions and the other factors like participant properties. Between the participant identity and the participant properties, the mutual information is relatively high; identity is statistically correlated with properties of the individuals. The mutual information between the participant properties is high differently for the different pairs; some different properties are statistically correlated with each other, others not. For the one-dimensional projection of the hand trajectory, the highest mutual information exists with the participant identity (except for the mutual information with the one-dimensional trajectory itself), indicating that the statistical correlation with the hand trajectory is highest for the identity. The factors that are correlated second highest with the participant properties are, in descending order, agreeableness, openness, body size, neuroticism, weight, time since last meal, conscientiousness, age, hours for sports, extraversion, health of the right arm or shoulder, profession, familiarity with the other subject and gender. The algebraic sign of the mutual information stays positive for these factors, which should be expected for this mathematical method. The mutual information of a factor with itself gives the highest value for the respective row and column; for each factor, the statistical correlation is strongest with itself. These diagonal values are quite different depending on the analysed factor, indicating a different amount of information that is available for the respective factor.

### 3.2. Supervised Learning

The results for the use of the Learning Vector Quantization Network with trialwise Cross-Validation are given in Table 4. As the network works with a chance factor, this is only one example; each use of the network leads to different results. However, the results presented here can be seen as archetypical. The factors, on which the Learning Vector Quantization Network was trained, are given in the second column. The first column separates them into interindividual factors (these are the determined properties of the participants, corresponding to between-subject designs, abbreviated in the table as “b”) and intraindividual factors (these are the experimental conditions, corresponding to within-subject designs, abbreviated as “w”). The proportion of wrong classifications (mean value for all partitions) is given in the third column. The number of partitions for the cross-validation is listed in the last column. For each partition, when it was used as test dataset, a cross-tabulation was performed. The mean value of the resulting *p*-values or, in other words, values of the asymptotic significance is given in the fourth column. Only when the network was trained to distinguish hand trajectories observed in trials with a platform from trajectories in trials without platform does the cross tabulation lead to *p*-values over 5%, so that no significant relation between the classification of the trajectories by the network and the presence of the platform could be proofed by the used methods. In the case of all other explored factors, the *p*-values were under the 5%-level, indicating a significant relation between the values of these factors found by the network and the real values. For the intraindividual factors, both the error rate and the *p*-values are lowest for the factor “side of the giver”.

The results for the participantwise cross-validation is that, after training, the network was assigning the values of the factors randomly to the trajectories; it was not able to recognise the participants’ properties from the trajectories. One problem here could be that the participants of our experiment have quite different properties, which means that there are only a few properties shared by a higher number of participants.

### 3.3. Unsupervised Learning

Looking at the 20-dimensional case, the results for a MANOVA with the factors “experimental condition” and “participant” as independent variables are given in Table 5. The asymptotic significance or *p*-value for the impact of the identity of the participant on the position of the neuroid, which specialised in the identification of a single subject, is under 5% for all partitions. (Only the mean value for training processes with all permutations of the choose of the testing partition is given in the table.)

When the impact of the single conditions and participant properties was evaluated using MANOVA, the model reduction led to a model with the remaining factors “gender”, “weight”, “sportiness”, “satiety”, “openness”, “conscientiousness” and “extraversion”. The results of a further MANOVA are given in Table 6.

In the case of a RANOVA, it was not possible to reduce the Repeated Measures Model in a way that the *p*-value for the correlation between one analysed factor with the position of the specialised neuroid was under 5% in all partitions.

## 4. Discussion

### 4.1. Multidimensional Scaling

As we have seen in Figure 5, hand trajectories of different participants are concentrated in different regions of the space, separated from the trajectories of other people, forming different clusters. In addition, for each participant, markers belonging to the role as giver or receiver, respectively, are concentrated in different parts of these clusters and separated from each other, forming two sub-clusters.

This kind of clustering leads to the conclusion, that the identity of the participant has a higher correlation with the hand trajectory than the experimental condition.

A change in the factor “side of the giver” causes a complete inversion of the trajectory. The effect of the other factors of the conditions can be considered to be smaller. It can be expected that the identity of the participant who produced a certain trajectory and his or her role as giver or receiver can be concluded from the position of the projection. The subdivision of the clusters indeed indicates that the same role of the subject (giver or receiver) leads to similar hand trajectories, a different role to dissimilar trajectories.

The analysis of a single experiment with its two participants leads to the same results, as shown in Figure 6. The division between different participants is even better visible.

As two different factors are mirrored by a clustering along two different dimensions, differences between trajectories caused by the respective other factor have another nature.

There is a bigger spreading of markers representing trials with a full beaker than for an empty beaker. Thus, a full beaker leads to a higher impact of the other factors.

The mutual information analysis (Figure 7) shows that the five factors of the experimental condition are independent from each other. As the choose of the factors for each trial was accidental, this is the expected result. The relatively high mutual information between the participant identity and the participant properties shows that an identification of a person and his or her properties are closely related. Some pairs of participant properties have a high mutual information, others not. The one-dimensional trajectory seems to be correlated most with the participant identity, followed by the participant properties in the sequence agreeableness, openness, body size, neuroticism, weight, time since last meal, conscientiousness, age, hours for sports, extraversion, health of the right arm or shoulder, profession, familiarity with the other subject, and gender. According to the definition of mutual information, it has to be highest for the comparison of a factor with itself. This is visible in our results. The value of the mutual information of a factor with itself depends on the total information content of the factor. The number of different values differs between the factors, which influences the information content of the factors. These differences are also visible in the results for the determination of the mutual information.

### 4.2. Supervised Learning

Trialwise cross-validation allowed for training the network to identify an experimental factor by trajectories. A correct classification needed training with trajectories from the test participant; it was not possible with participantwise validation. The participant can be identified by the position of the trajectory’s projection, as the correlation between trajectory and identity is highly significant (Table 4). This exactness of the identification of the participants is possible, as the projections of the trajectories for the same participant are close to each other but separated from the projections for others. The analysis confirms that the experimental conditions can be identified by a classification of the trajectory.

Individuality correlates most with trajectories. The conditions (except platform usage) also correlate with trajectories, especially for the side of the giver, overshadowed by individuality. The participant properties do not correlate with the trajectories in a way which could be separated from individuality.

### 4.3. Unsupervised Learning

The positions of the projections of the markers in the projection space are learned correctly by the Self-Organizing Map, connecting neuroid positions with experimental factors. Differences between trajectories caused by differences in the other factors are weaker than differences between trajectories caused by the individuality of the participants. This is only a first impression when interpreting the two-dimensional case visually; an exact analysis is done for the case of the 20-dimensional projection space: The results of the unsupervised learning confirm the higher impact of the participant identity on the hand trajectories, when compared to the impact of the condition. It is not possible to distinguish if the participant properties or identities were learned, or to divide the correlation of the properties with the trajectories from the correlation of the identity with the trajectories. Statistically relevant differences between hand trajectories are mostly caused by the individuality of the participants and not by definable properties of the participants or the experimental conditions. The analysis by means of unsupervised learning therefore confirms the findings, which were made when using supervised learning.

### 4.4. Answers

After our study, the questions asked at the beginning can be answered in the following way:

1. When the participants or some of the experimental conditions are similar or the same, the correspondent points in the projection space are contained in the same cluster or subcluster. This means that a matching between the clustering in the projection space on one side and the participant identity or some of the experimental conditions on the other side is possible. Therefore, most of the experimental conditions can be recognized by a classification of the resulting kinematic time courses.
2. Some differences between experimental conditions and a change of the participant identity were causing the projections of the resulting trajectories in the projection space to be contained in different clusters, indicating distinguishable behaviours. They were less related than trials with the same participant in the same condition, which is stressing that the differences are causing different behaviours. When the points in the projection space were classified by a Learning Vector Quantization Network, which was trained in a suitable way, it was possible to show a statistically significant relation between the result of the classification on one side and the participant identity or the experimental conditions on the other side. Therefore, different experimental conditions lead to results that are distinguishable by the data, also in the sense of the possibility of an automatic classification.
3. A hierarchy of the impact of different factors on the hand trajectories can be shown in the sense that individuality is the factor with the highest influence on the hand trajectories, followed by the experimental conditions, whereas the definable properties of the participants have no influence that could be separated from the influence of their individuality.

### 4.5. Outlook

As individuality seems to have the strongest correlation with hand trajectories, it could be difficult to find a general model of human hand-over movements. However, within one individual, the movements could be seen as more or less reproducible reactions on a given situation. With this background, it could be fruitful to explore the motion primitives underlying the hand motions of this individual. This could be done in other studies. For our experiment, a collection of human hand-over motions under standardised conditions was won, which can be used for further comparative studies. The motion primitives found in those studies could be transferred on an artificial system—for example, the shadow hand (see, for example, Röthling et al. [20]).

The only information source about the motions used in this study are the positions of the markers attached to the hands of the participants. Further studies can include information about the forces applied to the object in the hand-over process. For this, the plastic cans used in this study could be replaced by an object, which contains the necessary sensory equipment. A device, which could be used for this purpose, is the so-called iObject, as it was developed by Kõiva et al. [33]). From the study presented here, we know which factors could have a statistically significant correlation with the hand trajectories. When designing a study using the iObject, we could have a closer look at these factors, hoping to find a stronger correlation, when data about force distributions are added to the motion data that is analysed here.

## Figures and Tables

**Table 1:** Physical properties of the analysed participants.

Factor	Unit								
identity	number	1	2	3	4	5	6	9	10
gender	nominal	male	male	female	male	male	female	male	female
age	years	28	25	26	24	73	72	25	44
body size	m	1.72	1.75	1.65	1.76	1.82	1.70	1.76	1.64
weight	kg	72.0	68.0	70.0	65.0	77.0	58.5	72.0	60.0
profession	nominal	student	student	student	student	pensioner	housekeeper	student	staff
sportiness	hours	4.0	5.0	1.5	1.5	2.0	1.0	7.0	5.0
citizenship	nominal	German	German	German	German	German	German	German	German
satiety	hours	0.2	2.0	2.0	2.5	3.0	3.0	2.0	5.0
familiarity	1 to 5	1	1	1	1	5	5	1	1
health	nominal	no	no	no	no	no	yes	no	yes

**Table 2:** Results of the Big Five Test for the analysed participants.

Factor	P1	P2	P3	P4	P5	P6	P9	P10
openness	05	53	12	84	70	24	76	76
conscientiousness	13	58	47	69	69	58	94	64
extraversion	48	37	09	53	37	48	42	59
agreeableness	74	32	00	83	50	50	50	94
neuroticism	27	22	22	18	37	22	32	11

**Table 3:** Parameters of the multidimensional scaling.

Parameter	Value
Start	cmdscale
Replicates	1
MaxIter	200
TolFun	0.0001
TolX	0.0001

**Table 4:** Supervised learning: Results of trialwise cross-validation.

Design	Factor	Error	Asympt. Sign.	N
w	side of the giver	0.0988	0.0000	12
b	extraversion	0.0303	0.0004	44
b	familiarity	0.0079	0.0020	43
b	body size	0.0323	0.0006	45
b	neuroticism	0.0317	0.0009	43
b	age	0.0242	0.0012	45
b	health	0.0301	0.0011	43
b	profession	0.0364	0.0009	45
b	conscientiousness	0.0303	0.0012	45
b	weight	0.0323	0.0013	45
b	hours since last meal	0.0283	0.0016	45
b	agreeableness	0.0343	0.0014	44
b	openness	0.0222	0.0023	45
b	hours for sports	0.0342	0.0016	45
b	gender	0.0281	0.0045	45
b	participant identity	0.0419	0.0066	43
w	kind of handover	0.3327	0.0013	4
w	content of the beaker	0.2984	0.0059	7
w	beaker size	0.3427	0.0055	4
w	platform	0.5403	0.1727	2

**Table 5:** Unsupervised learning: Results of MANOVA.

Factor	Statistic	Mean Asympt. Sign. (p)	N
(intercept)	Pillai	0.2030	8
(intercept)	Wilks	0.2030	8
(intercept)	Hotelling	0.2030	8
(intercept)	Roy	0.2030	8
condition	Pillai	0.3927	8
condition	Wilks	0.3480	8
condition	Hotelling	0.3063	8
condition	Roy	0.0011	8
participant	Pillai	0.0065	8
participant	Wilks	0.0024	8
participant	Hotelling	0.0005	8
participant	Roy	0.0000	8

**Table 6:** Unsupervised learning: MANOVA, participant properties.

Factor	p_partition 1_	p_partition 2_	p_partition 3_
(intercept)	3.86E-03	1.36E-04	1.62E-05
gender	5.93E-19	1.21E-05	7.00E-16
weight	1.57E-04	5.36E-03	6.28E-09
sportiness	8.63E-16	7.76E-11	8.36E-09
satiety	1.55E-15	4.98E-07	1.84E-12
openness	6.21E-15	1.13E-06	3.99E-11
conscientiousness	4.93E-10	2.32E-02	4.10E-07
extraversion	1.11E-06	3.29E-07	1.01E-10

## Abbreviations

The following abbreviations are used in this manuscript:

MANOVA	multivariate analyses of variance
RANOVA	repeated measures analyses of variance
